# Clonal hematopoiesis and atherosclerosis

**DOI:** 10.1172/JCI180066

**Published:** 2024-10-01

**Authors:** Ohad Oren, Aeron M. Small, Peter Libby

**Affiliations:** 1 Division of Cardiology, Massachusetts General Hospital, Harvard Medical School, Boston, Massachusetts, USA.; 2 Division of Cardiovascular Medicine, Department of Medicine, Brigham and Women’s Hospital, Harvard Medical School, Boston, Massachusetts, USA.

## Abstract

Clonal hematopoiesis of indeterminate potential (CHIP) has emerged as a previously unrecognized, potent, age-related, and common risk factor for atherosclerosis. Somatic mutations in certain known leukemia driver genes give rise to clones of mutant cells in peripheral blood. The increased risk of developing hematologic malignancy does not, on its own, explain excess mortality in individuals with CHIP. Cardiovascular disease accounts for much of this gap. Experimental evidence supports the causality of certain CHIP mutations in accelerated atherosclerosis. CHIP due to mutations in different driver genes varies in their promotion of atherosclerotic events and in the region of augmented atherosclerotic involvement. For example, CHIP due to mutations in *DNMT3a* appears less atherogenic than CHIP that arises from *TET2* or *JAK2*, forms of CHIP that incite inflammation. The recognition of certain CHIP mutations as promoters of atherosclerotic risk has opened new insights into understanding of the pathophysiology of this disease. The accentuated cardiovascular risk and involvement of distinct pathways of various forms of CHIP also inform novel approaches to allocation of targeted therapies, affording a step toward personalized medicine.

Clonal hematopoiesis of indeterminate potential (CHIP) is defined as the presence of a somatic mutation in a leukemia driver gene involving blood cells of an individual lacking any known hematologic malignancy, cytopenia, dysplasia, or other recognized clonal disease. Diagnosis of CHIP specifically requires exclusion of hematologic neoplasia such as myelodysplastic syndrome (MDS). To classify as CHIP, the driver mutation must have a variant allele frequency (VAF) of at least 2% in the peripheral blood. Recognition of CHIP arose from a seemingly unrelated quest to elucidate the cellular origins of leukemia. When CHIP was discovered and its associations with clinical outcomes were illuminated, an unexpected finding emerged, igniting substantial scientific interest and spurring nontraditional cross-specialty collaborations. While the presence of CHIP conferred a 30%–40% higher risk of total mortality, the increased rate of hematologic malignancies fell short of accounting for the excess deaths (1). Rather, higher cardiovascular death explained most of the mortality increase, and additional work revealed that the presence of CHIP predicted up to a two-fold higher incidence of coronary artery disease and a four-fold increase in early onset myocardial infarction (1). Thus, CHIP has emerged as a previously unrecognized, potent, and common risk factor for atherosclerosis. These intriguing epidemiological correlations triggered a series of in-depth biologic investigations into the molecular underpinnings of atherosclerosis in CHIP.

## CHIP: historical framework

The roots of the CHIP discovery go back to 2012, when several lines of evidence converged to implicate clonal hematopoiesis driven by somatic mutations in certain known leukemia driver genes as a harbinger of acute myeloid leukemia (AML). A pivotal investigation by Busque et al. uncovered the presence of *TET2* mutations in approximately 5% of healthy elderly women with nonrandom X chromosome inactivation, underscoring the capacity of *TET2*-mutated hematopoietic stem cells (HSCs) for self-renewal and clonal expansion in a host free of hematopoietic malignancies (2). Building upon this study, Shlush et al. demonstrated the persistence of *DNMT3A* mutations in HSCs of patients with AML whose blasts expressed both *DNMT3A* and *NPM1* mutations, suggesting that preleukemic clones undergo stepwise acquisition of genetic lesions as they develop into leukemia (3). Corces-Zimmerman et al. further showed that, in the evolution of AML, mutations in genes involved in global chromatin changes (i.e., *DNMT3A* and *IDH1/2*) occur early, while mutations in “proliferative” genes (i.e., *FLT3*) tend to occur late (4). Together, these studies identified *DNMT3A* as an early leukemia-initiating gene and, more broadly, established CHIP as an asymptomatic potential precursor of myeloid diseases, analogous to monoclonal gammopathy of undetermined significance as a potential precursor of multiple myeloma and monoclonal B cell lymphocytosis as a potential precursor of CLL.

**Figure 1:**
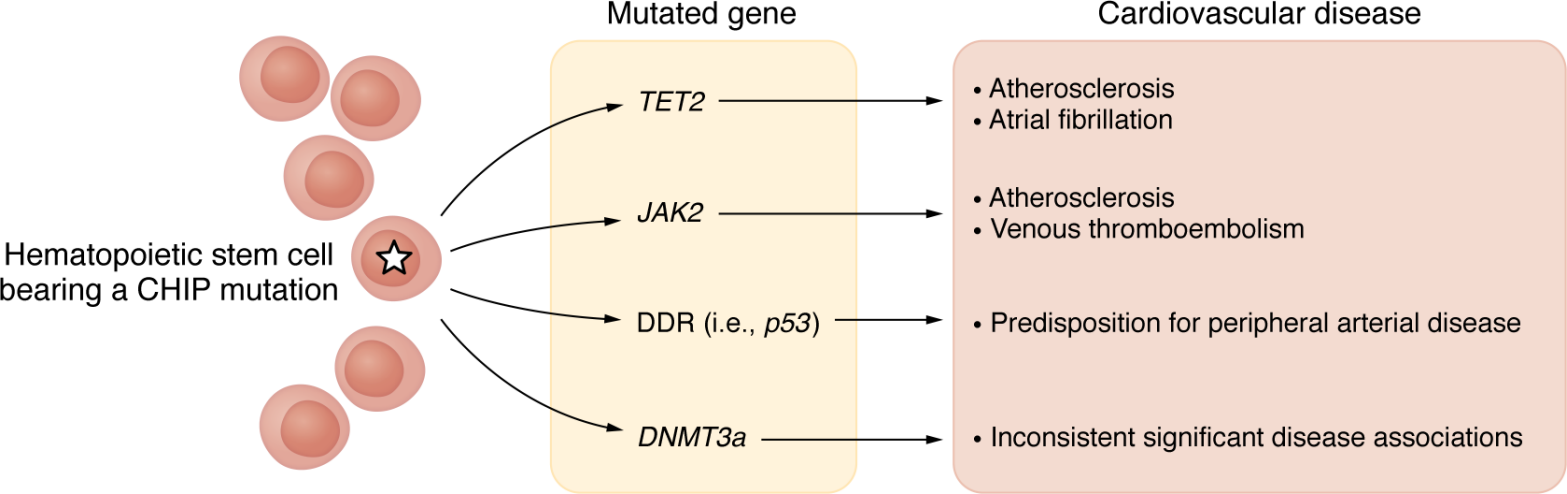
Different CHIP mutations have varied manifestations and degree of cardiovascular risk. Diversity in risk and manifestations among common CHIP mutations. Associations between specific CHIP mutations and cardiovascular disease including not only atherosclerosis but atrial fibrillation, peripheral artery disease, and venous thromboembolism.

These investigations that probed the origins of leukemia did not address the prevalence of CHIP in the general population, its clinical associations, natural history, and full genetic architecture. Further study leveraging exome-sequencing data derived from population-based genetic association studies expanded this initial knowledge (1, 5, 6). Such work led to the pivotal discovery that CHIP appears to stem from somatic mutations in a limited number of known leukemia driver genes (Figure 1). Loss-of-function (LOF) mutations in genes encoding DNA methylation enzymes (*DNMT3A, TET2*) accounted for approximately two-thirds of CHIP, followed by chromatin regulation (i.e., *ASXL1*) and splicing (i.e., *SF3B1*, *SRSF2*, *U2AF1*) gene mutations, in a study of blood-derived sequence data from 2,728 individuals from The Cancer Genome Atlas (TCGA) (5).

Subsequent analyses have shed light on the fundamental relationship between age and CHIP. While less than 1% of healthy individuals under the age of 40 had CHIP, at least 10%–20% of those age 70 or older harbored a clone, defined as a VAF of 2% or more (1, 5). In addition, while individuals as young as 25 years could have *DNMT3A* mutations, spliceosome gene mutations, such as *SF3B1* and *SRSF2*, occurred primarily in those older than 70 years of age, suggesting that the emergence of the latter group of mutations in later years accounts for the increased incidence of clonal disorders in older individuals (5).

Analyses of CHIP in various biobanks and cohorts highlighted that the methods used for clone quantification can substantially influence the rate of detection. Sensitivity for detection of driver mutations is a function of sequencing depth. Whole-exome sequencing (WES) efficiently detects larger clones (>3%), while very small clones require deeper coverage, such as with targeted gene panels. Ultrasensitive sequencing methods allow the identification of particularly small clones (variant allele frequencies as low as 0.001), but the natural history and clinical implications of such tiny clones remain unknown (7, 8).

Once studies of CHIP revealed that atherosclerotic cardiovascular disease (ASCVD) contributed importantly to increased morbidity and mortality, experimental studies probed the causality of CHIP in atherogenesis. Pivotal work by Fuster et al. in atherosclerosis-prone, low-density lipoprotein receptor–deficient (*LDLR*-deficient) mice whose bone marrow was reconstituted with *Tet2*-deficient cells showed a significant increase in atherosclerotic plaque size relative to wild-type control mice (9). Activation of NLRP3, a multiprotein inflammasome complex, and its downstream effects on IL-1β maturation appeared to cause this exacerbated atherosclerosis, as the administration of an NLRP3 inhibitor to these mice decreased atherosclerotic plaque size, decreasing the inflammatory impact of Tet2 mutant cells (9). Simultaneous studies by Jaiswal et al. also showed accelerated atherosclerosis in mice with myeloid *Tet2* deficiency in a gene-dosage–dependent manner and evidence for augmented expression of proinflammatory mediators including IL-1 and IL-6 in peripheral blood and in mutant leukocytes (10). Indeed, a study of approximately 98,000 individuals of diverse ancestries leveraging high-coverage whole-genome sequencing (WGS) showed an association between TET2 CHIP and increased circulating IL-1β (8). Studies of JAK2 V617F myeloproliferative neoplasms (MPNs) demonstrated that mice expressing Jak2V617F had higher propensity for neutrophil extracellular trap (NET) formation and exhibited augmented venous thrombosis. Ruxolitinib, a JAK/STAT signaling inhibitor, ameliorated this finding (11). Mice with myeloid Jak2VF show increased arterial thrombosis driven by platelet activation and elevated hematocrit (12). Experimental observations suggest that *Jak2*^VF^ and accentuated NET formation aggravate intimal injury implicated in superficial erosion, a cause of about a quarter of acute coronary syndromes (13).

Further, studies of *p53* mutations in CHIP illustrated the competitive survival advantage of hematopoietic stem and progenitor cells (HSPCs) of mice expressing mutant p53 (14). Mechanistically, mutant p53 proteins interact with EZH2, a histone methyltransferase, augmenting the latter’s association with chromatin and consequently leading to higher levels of H3K27me3, an epigenetic modifier associated with increased proliferation (14).

While CHIP driver mutations can accelerate atherogenesis via perturbation of myeloid cell activity, the reverse might pertain as well. Thus, atherosclerosis and attendant risk factors may promote HSC proliferation as well as the onset of CHIP and its expansion. For example, hyperlipidemia may accelerate age-related development of CHIP. Indeed, computational evolutionary dynamic studies have concluded that HSC proliferation rates increase in patients with cardiovascular disease (15). Consistent with these findings, *Apoe^−/−^* mice showed significantly faster increases in the fraction of *Tet2^−/−^* in the bone marrow under conditions of increased HSC proliferation (15). Some investigations, however, have not borne out the conclusions of this mathematical modeling. A study of atherosclerosis-prone LDLR-deficient (*Ldlr^−/−^*) mice with a small proportion of *Tet2*-deficient HSPCs showed no significant changes in the degree of clonal expansion when the mice consumed a chow diet versus a high-fat/high-cholesterol diet. (9). Similarly, understanding of multidirectional interactions among HSC proliferation, CHIP, and atherosclerosis is needed to define further the associations between exposures associated with chronic increases in HSC division (i.e., smoking, sleep deprivation, psychological stress) and various disease states (i.e., diabetes, HIV, heart failure) and CHIP (16).

## Biology and genetic mechanisms underlying CHIP

### Biologic underpinnings of CHIP driver mutations.

Most individuals with CHIP have a single driver mutation, and the majority of these (upwards of 75%) take place in one of three genes: *DNMT3A, TET2*, or *ASXL1* (8) (Figure 1). Approximately 15% of individuals with CHIP have driver mutations in one of a handful of additional genes, including *JAK2, PPM1D, SF3B1, SRSF2,* and *TP53*. Some individuals have clonal hematopoiesis without a defined driver mutation (~40%), such as in the case of mosaic chromosomal alterations (mCAs), structural somatic alterations of peripheral leukocyte chromosomes (17).

The three most common CHIP driver mutations, *DNMT3A, TET2*, and *ASXL1*, encode epigenetic regulators that alter gene transcription via DNA or histone methylation. Transcriptional stimulation of inflammation by certain CHIP mutations contributes mechanistically to increased cardiovascular risk. *TET2* encodes a tumor suppressor gene that classically prevents development of myeloid malignancies. *TET2* LOF appears to promote atherosclerosis primarily via enhanced transcription and NLRP3-mediated posttranslational processing of the inflammatory cytokine IL-1β. Accordingly, *TET2* LOF macrophages treated in culture with a cocktail of oxidized LDL, TNF, and IFN-γ (to mimic conditions within the atherosclerotic plaque) develop marked increases in IL-1β (9). Recent data further support a synergistic effect of chronic IL-1β exposure on maintaining fitness of *TET2* LOF cells, whereby IL-1β itself promotes epigenetic upregulation of self-renewal genes in *TET2*-deficient HSCs (18). Individuals with *TET2* CHIP also have higher circulating levels of other cytokines mediated by the NLRP3 inflammasome, including IL-8 (10), and pharmacologic inhibition of IL-6 signaling mitigates *TET2*-mediated cardiovascular risk in a *Tet2* clonal hematopoiesis murine model (19). Importantly, murine models of *TET2* LOF demonstrate larger atherosclerotic lesions in both TET2-homozygous and -heterozygous hematopoietic cells (9, 10).

*TET2* and *DNMT3A* each regulate DNA methylation. However, epigenome-wide association analysis clarifies that these two genes promote HSC renewal by opposite actions; *TET2* LOF generally results in DNA hypermethylation, whereas *DNMT3A* LOF results in DNA hypomethylation (20). Despite different methylation patterns, *TET2* or *DNMT3A* CHIP may both drive atherosclerosis by boosting inflammation. Rauch et al. found that homozygous *Dnmt3a* LOF in murine myeloid cells increased atherosclerosis to an extent similar to that of *Tet2* LOF (21). Additionally, bone marrow–derived myeloid cells from *Dnmt3a* LOF mice exhibit a gene expression profile similar to that of *Tet2* LOF, including increased expression of the key inflammatory cytokines IL-1β and IL-6. Interestingly, *Dnmt3a* LOF augmented atherosclerosis only when homozygous, in contrast with findings with *Tet2* LOF. In humans, CHIP due to *DNMT3A* shows a weaker association with atherosclerosis than TET2. Further, genetically reduced IL-6 signaling does not mitigate *DNMT3A*-mediated cardiovascular risk (22).

In contrast to *TET2* and *DNMT3A*, where NLRP3 inflammasome activation appears predominant as a mechanism that drives accelerated atherosclerosis, recent data suggest that *ASXL1* and *JAK2* CHIP provoke activation of the AIM2 inflammasome (Table 1). A recent human genetic study using data from the UK Biobank (UKB) demonstrated that genetically predicted increased expression of *AIM2* (encoding an initiating protein of the AIM2 inflammasome) is associated with cardiovascular risk only in the presence of *ASXL1* or *JAK2* CHIP (23). A murine model of *ASXL1* CHIP similarly supports AIM2 inflammasome activation, but not NLRP3 inflammasome activation, after lipopolysaccharide challenge (24). So far, murine models of *ASXL1* CHIP have not shown increased atherosclerosis. However, these experiments may be of too short duration to settle this question.

*JAK2* encodes a nonreceptor tyrosine kinase that when mutated (*JAK2*p.V617F [*JAK2^VF^*]) is associated with a host of MPNs including essential thrombocythemia, polycythemia vera, and myelofibrosis. *JAK2^VF^* CHIP can occur at a younger age and portends the highest cardiovascular risk of established CHIP driver mutations (8). *JAK2^VF^* mutations promote the formation of procoagulant NETs, which facilitate the thrombotic complications characteristic of morbidity in *JAK2^VF^* myeloproliferative disorders (11, 24). NETs may contribute to the morbidity of *JAK2^VF^* CHIP, with increased incidence of thrombosis among these individuals (11). Like *ASXL1*, *JAK2 ^VF^* CHIP also drives atherosclerosis by activating the AIM2 inflammasome. Atherosclerotic lesions are increased in Jak2VF CHIP mice and show increased AIM2 expression, while genetically predicted *AIM2* gene expression in humans is associated with increased cardiovascular risk only among individuals with *JAK2^VF^* CHIP (25, 26).

The biologic mechanisms explaining enhanced atherosclerotic risk among other CHIP driver mutations are less well defined. *TP53* and *PPM1D* encode DNA damage repair (DDR) proteins and portend increased atherosclerotic risk in both coronary and peripheral arteries (27) (Figure 1). Recent experimental evidence demonstrates that *TP53* CHIP’s association with accelerated atherosclerosis results from enhanced clonal macrophage expansion within the atherosclerotic plaque, rather than inflammasome activation. Accordingly, individuals with DDR CHIP do not manifest increased inflammatory cytokines (e.g., IL-6 and IL-1 β) (8). Atherosclerosis-prone mice transplanted with *Trp53^–/–^* hematopoietic cells showed an approximately 40% increase in aortic plaque size relative to *Trp53^+/+^* mice as well as accumulation of intraplaque macrophages, with no effect on proinflammatory cytokines or the NLRP3 inflammasome (27).

## Germline genetic variation underlying CHIP pathogenesis

Studies of germline genetic variation predisposing to incident CHIP provide additional opportunity for understanding the biologic basis of atherosclerosis in CHIP. To date, several genome-wide association studies (GWAS) have characterized germline variation predisposing to CHIP. A study of 65,405 individuals in TOPMed (8), 200,454 individuals from the UKB (28), and 45,510 individuals from Iceland (deCODE) (29) identified 25 unique loci with implicated mechanisms spanning DDR, HSC migration, telomere length, and MPN development. The most strongly replicated GWAS locus favoring CHIP is *TERT*, which encodes telomere reverse transcriptase, a protein that helps lengthen telomeres. Interestingly, and somewhat paradoxically, while shorter telomere length has a strong causal association to atherosclerosis (30), Mendelian randomization also demonstrates that genetically imputed longer telomeres associate causally with CHIP (30). Nakao and colleagues hypothesized that longer telomeres may hasten CHIP acquisition, but once an HSC acquires CHIP, telomere shortening accelerates, explaining the causal associations observed between both longer telomeres and incident CHIP as well as shorter telomeres, CHIP, and increased cardiovascular risk (30).

Another approach to understanding germline variation contributing to the development of CHIP, the passenger-approximated clonal expansion rate (PACER), considers an individual’s burden of somatic mutations (“passengers”) by sequencing whole-blood DNA (31). Weinstock and colleagues performed a GWAS of PACER and identified a common polymorphism in a promoter for the gene *TCL1A*, which encodes a protein previously implicated in lymphoid malignancies (31). The fine-mapped lead variant in the promoter for *TCL1A* slows the CHIP expansion rate, which — in addition to in vitro data — points to this gene or its protein as a treatment target for CHIP. Intriguingly, TCL1A led to in vitro HSC expansion in *TET2* and *ASXL1* mutants, but not in the most common variant, *DNMT3a*.

### Clonal controversies.

Despite wide replication of the association between CHIP and incident cardiovascular disease, some studies have not found this link, which appears to depend on the CHIP driver mutation. A notable example is the weaker association between *DNMT3A* CHIP and coronary or peripheral artery disease in the full UKB (27), which contrasts with earlier findings including those by Jaiswal and colleagues in 2017 (10). While other CHIP driver mutations, including *TET2*, associate with adverse cardiovascular outcomes among individuals with prevalent atherosclerosis, *DNMT3A* seems not to confer the same risk, although some studies did show a weak association with elevated incident coronary heart disease (32,33). The contributions of *DNMT3A* to cardiovascular risk require further study, especially in regard to disease processes beyond atherosclerosis, such as cardiac fibrosis and heart failure (30). In another example of a null association between CHIP and cardiovascular outcomes, Stacey and colleagues found no association between all CHIP and cardiovascular disease in a meta-analysis of UKB and deCODE samples (29). However, the authors agglomerated CHIP, combining multiple types of somatic mosaicism together, including classic driver mutations such as *TET2* and *DNMT3A*, but also mosaic loss of chromosome Y and other mCAs. Several prior studies established that autosomal mCAs are not associated with cardiovascular disease (34, 35). These investigators also hypothesized that the increased risk of cardiovascular disease in clonal hematopoiesis arose from confounding by cigarette smoking. However, smokers and nonsmokers have similar CHIP prevalence in the overall UKB, refuting this suggestion (36). The accurate determination of CHIP in large populations requires a well-validated quality control pipeline. CHIP can be ascertained either by using WGS or WES from large population studies (relatively lower sequencing depth) or by targeted gene panels (relatively greater sequencing depth). The sensitivity to detect small clones, and then to accurately describe relationships between CHIP and disease, correlates positively with sequencing depth. The practical challenges to sequencing large populations with targeted gene panels underscores the importance of adopting validated pipelines to call CHIP accurately from lower sequencing depth platforms such as WGS/WES, similar to efforts like those developed by Vlasschaert and colleagues and applied to data from the UKB and All of Us (37). Rigorous calling of CHIP requires considerable care. Pitfalls include sequencing artifacts and confounding by germline mutations (37).

## CHIP’s clinical implications

More than 10% of septuagenarians have at least one CHIP driver mutation and, depending on the driver mutation, may have an up to four-fold increased risk of cardiovascular events compared with unaffected, healthy individuals (10). The relative contribution to cardiovascular risk imparted by CHIP among older individuals does not depend on traditional cardiovascular risk factors such as smoking, hypercholesterolemia, and diabetes and may even rival the risk imparted by these common comorbidities (38). As the population ages, most clinicians will almost certainly routinely encounter patients in practice with recognized or unrecognized CHIP. Two important questions arise. First, how can we best manage cardiovascular risk among individuals with established CHIP? And second, given the common occurrence of CHIP in older age and relatively high risk of cardiovascular events among those with CHIP, is there a role for screening among asymptomatic, otherwise healthy individuals?

A small but growing number of clinics consider cardiovascular risk assessment for individuals with CHIP. For example, at the Brigham and Women’s Hospital, we see patients referred for newly established CHIP diagnosed in the context of workup for another condition or self-referred individuals. Common pathways by which a patient might be referred include diagnosis by multi-gene sequencing panels performed either for evaluation of cytopenia without overt myelodysplasia or as screening for recurrence of malignancy after bone marrow transplant or in DNA analysis of peripheral blood as a matched control specimen for DNA sequencing of tumor specimens (39). Importantly, no prospective clinical trials guide decision making for these individuals. Rather, we educate patients about CHIP and cardiovascular risk and have an informed discussion about addressing modifiable cardiovascular risk factors, including hypercholesterolemia, smoking, diabetes, and obesity. We also consider the specific driver mutation and VAF in their relative propensity to cause disease. Higher VAFs or CHIP driver mutations that appear to confer particular elevated cardiovascular risk (e.g., *TET2* or *JAK2^VF^*) prompt us to recommend a more aggressive preventive strategy, including more intensive LDL lowering. We engage in shared decision making regarding the addition of aspirin as a primary prevention strategy for individuals with *JAK2^VF^* CHIP, given the prothrombotic nature of these mutations. For individuals with *TET2* CHIP, we discuss empiric vitamin C supplementation, a cofactor for TET2 activity (40).

Despite the common occurrence of CHIP mutations among the older general population, we do not yet recommend routine screening. As we currently lack a targeted treatment strategy for this population and prospective trials, we judge it premature to advise healthy, asymptomatic patients to consider testing. Direct-to-consumer testing options are increasingly available to patients, and the general practitioner may soon more frequently encounter patients with incidentally discovered CHIP. Further, targeted therapeutics acting against inflammation may become available as a treatment option to mitigate cardiovascular risk for the appropriate CHIP driver mutations. We should reassess the appropriateness of routine CHIP screening as treatment options mature.

## Therapeutic vistas: targeting inflammation in *TET2* and *JAK2* CHIP

**Figure 2:**
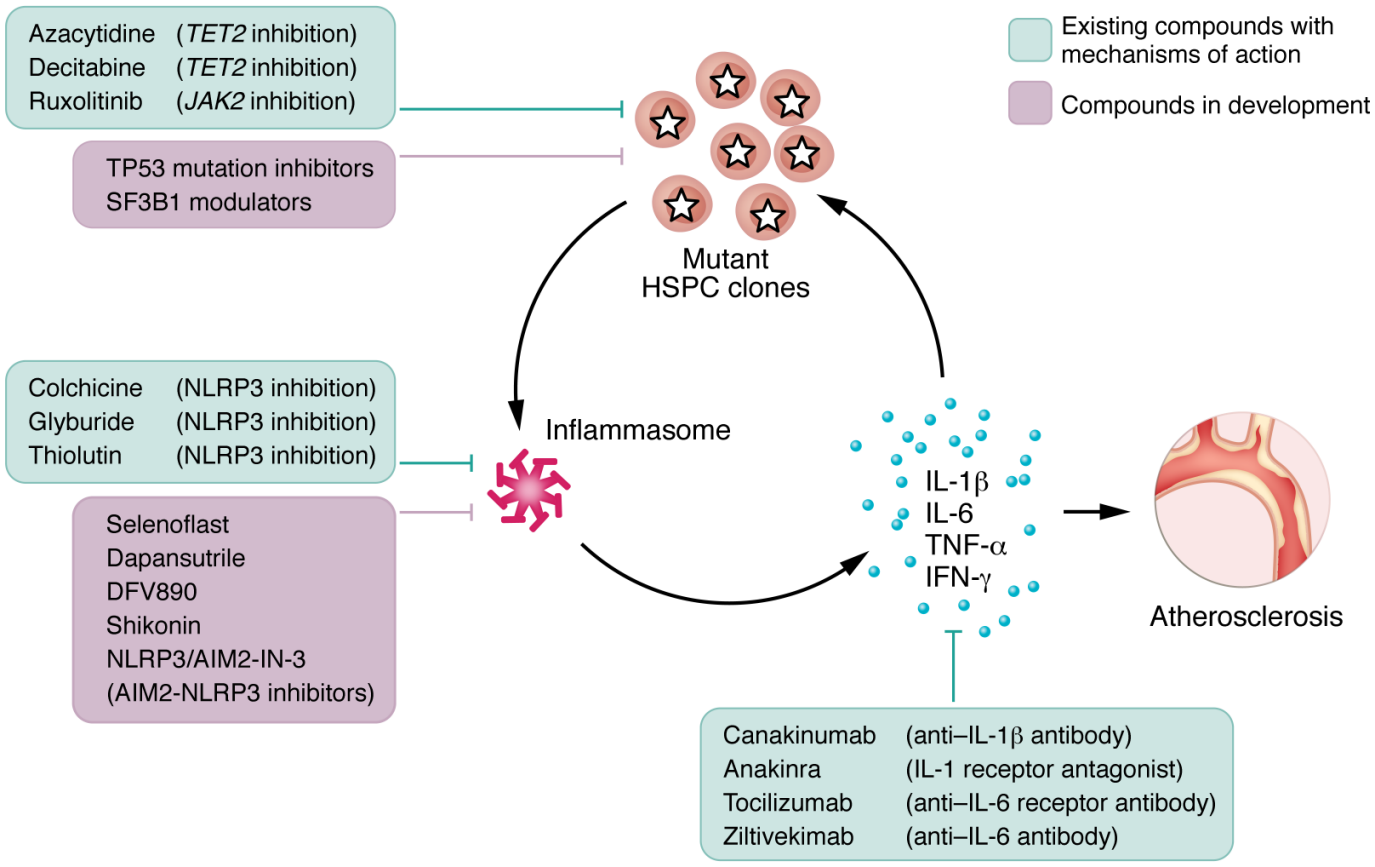
Therapeutic possibilities to suppress progression of certain CHIP mutations and downstream atheroprotection. Existing drugs and compounds in development that may suppress CHIP clones or downstream inflammatory cascade, with the potential to inhibit atherogenesis in CHIP.

Although we currently lack available interventions proven to reduce cardiovascular risk for individuals with CHIP, the search for a targeted preventive and therapeutic agent continues (41) (Figure 2). Given that mutant HSPCs, and in particular those harboring *TET2* variants, lead to elevated inflammatory responses in myeloid cells, inhibition of inflammatory mediators merits consideration. One method of ascertaining this relationship is through subgroup analyses of completed clinical trials of antiinflammatory agents. One such exploration leveraged Canakinumab Anti-inflammatory Thrombosis Outcomes Study (CANTOS), a randomized clinical trial of canakinumab, an anti–monoclonal antibody IL-1β in patients with a history of myocardial infarction and above median high-sensitivity C-reactive protein (hsCRP). In CANTOS, patients who received canakinumab (150 mg) experienced a 15% relative risk reduction in the combined rate of myocardial infarction, cardiovascular-related death, and stroke, a significant benefit equivalent to that achieved with PCSK9 inhibition (an LDL-lowering strategy approved for individuals at high risk for ASCVD) (42). In CANTOS, greater IL-6 or CRP reductions correlated with lower event rates (43), again implicating innate immunity in ischemic heart disease. In an exploratory substudy of CANTOS, patients with *TET2* CHIP who received canakinumab had lower major adverse cardiovascular events, in contrast with those with *DNMT3A* CHIP who showed no such effect (44). That finding supports the notion that *TET2* LOF mediates its proatherosclerotic effects via IL-1β and IL-6 signaling, inhibition of which may decrease atherogenesis and the consequent development of cardiovascular events, a notion that requires prospective testing.

Randomized controlled trials of the antiinflammatory agent colchicine in patients with history of myocardial infarction demonstrated a significantly lower risk of ischemic cardiovascular events compared with placebo (45, 46). An analysis of the results by clonal hematopoiesis status would hold particular interest when possible and should be prespecified in future cardiovascular studies with colchicine and other antiinflammatory interventions. Alternative compounds that similarly antagonize cellular mediators in the inflammatory cascade merit consideration for therapy in patients with CHIP mutations that augment IL-1β and IL-6. Such interventions could include antagonists of the inflammasome or of IL-6. In support of this concept, Mendelian randomization studies show that genetic loci implicated in immune system function (i.e., IL-6R, IL-1F10) associate with higher rates of coronary artery disease. In a phase 2 clinical trial of ziltivekimab (47), an anti–IL-6 ligand human monoclonal antibody, individuals with high cardiovascular risk who received the drug showed substantial reductions in biomarkers of inflammation and thrombosis, including hsCRP, fibrinogen, and serum amyloid A. These results helped inform the design of the ongoing ZEUS trial, a cardiovascular outcomes trial that will evaluate the effects of ziltivekimab in patients with established cardiovascular disease (48) in a broader attempt to quantify the cardiovascular benefits afforded by the reduction of residual inflammatory risk. Use of a humanized anti–IL-6 receptor monoclonal antibody, tocilizumab, in patients with rheumatoid arthritis consistently elevates atherogenic lipoproteins (49), hence, the importance of understanding the lipid and metabolic interactions of compounds that suppress systemic cytokines implicated in atherogenesis.

Modulation of inflammasome activity offers another possible strategy for amelioration of atherogenesis in individuals with *TET2* CHIP (Figure 2) (50–53). An ongoing phase Ic randomized clinical trial (GC43343; https://forpatients.roche.com/en/trials/cardiovascular-disorder/coronary-artery-disease/a-phase-ic-multicenter--randomized--double-blind--placebo-contro.html) is studying the use of selnoflast, an NLRP3 inhibitor, in patients with coronary artery disease and elevated hsCRP with a substudy evaluating the specific effects in subjects with pathogenic *TET2* variants. This trial will assess the effects of selnoflast on biochemical surrogates hsCRP and IL-1β. A pivotal consideration addressed in the trial is the drug’s safety and, in particular, its effects on immune function. An additional study, a phase IIa clinical trial (ClinicalTrials.gov NCT06097663), is assessing the efficacy, safety and tolerability of DFV890, also an NLRP3 inhibitor, and MAS825, a bispecific IL-1β/IL-18 monoclonal antibody (53). This multicenter study randomized approximately 28 individuals with known coronary artery disease and *TET2* or *DNMT3a* CHIP to different combinations of DFV890, MAS825, and placebo, administered for 12 weeks, with a 30-day follow up. The study will evaluate the effect of therapy on markers of inflammation, including IL-6 and IL-18. Other inflammasome inhibitors including dapansutrile have entered clinical development. Since NLRP3 inflammasome inhibition does not block the production of IL-1β via other inflammasomes, NLRP3 inhibition might interfere with host defenses less than inhibitors of IL-1β. Suppression of the AIM2 inflammasome could confer cardiovascular benefit, particularly in individuals with *JAK2* CHIP. Synthetic oligonucleotides that antagonize AIM2 are currently under development (54).

Yet another potential therapeutic vista includes the use of CHIP-mutation–specific modulators (Figure 1). Azacytidine and decitabine, two hypomethylating agents approved for patients with myeloid malignancies, show greater hematologic response rates in AML patients with *TET2* mutations compared with those with wild-type *TET2* (55). Such enhanced sensitivity to hypomethylation by *TET2* mutant cells likely results from perturbed downstream genomic methylation and protein expression. Whether hypomethylating agents disproportionately alter inflammatory pathways intrinsic to atherogenesis in individuals with *TET2* mutation is unknown, but this strategy has key cardioprotection implications in a population enriched in cardiovascular risk factors and diseases. Another approved drug with CHIP mutation–inhibiting properties, fedratinib, exerts therapeutic benefit in MPNs by way of its *JAK2* inhibition (56). The motivation to inhibit *JAK2* signaling to limit atherosclerosis stems from studies showing that constitutively activating *JAK2* mutants lead to biased myelopoiesis, which links to the development of ASCVD. Investigators have therefore studied the effects of *JAK2* inhibition with fedratinib in *Apoe^–/–^* mice who consumed an atherogenic diet (56). Fedratinib led to expected reversals in neutrophilia, monocytosis, and HSPC expansion as well as to substantial reductions in aortic atherosclerosis when compared with mice who were not treated with fedratinib (56). Ruxolitinib, an inhibitor of JAK1 and JAK2, is an FDA-approved drug used in patients with rheumatoid arthritis, albeit with a black box warning for cancer and cardiovascular disease. In male rabbits who consumed a high-fat diet, ruxolitinib significantly decreased atherosclerotic plaque size, an effect potentially mediated by the substantial reductions in IL-6, IL-1β, IFN-γ, and TNF-α.

## Future directions and conclusions

In just a decade from its first description, CHIP has emerged as a novel, common, and in the case of certain forms, potent age-related cardiovascular risk factor. The study of CHIP has offered previously unsuspected opportunities for elucidating novel mechanistic insights into disease pathophysiology as well as the intersection in the biology of immunity, inflammation, cancer, and atherosclerosis.

For the cardiovascular clinician, scientist, and trialist, CHIP provides an opportunity for enriching the definition of cardiovascular risk and, as sequencing technologies advance, its incorporation into trials offers novel possibilities to personalize the deployment of antiatherosclerotic and cardioprotective effects of antiinflammatory and immune-modulating compounds, which may offer particular value in individuals with specific CHIP mutations, mutation combinations, and high allele frequencies.

Yet despite the obvious gains in its understanding, the magnitude of cardiovascular disease potentiation encountered with CHIP as well as factors associated with particularly heightened cardiovascular risk require continued investigation. The community should aim to translate CHIP detection into clinically meaningful improvements in cardiovascular health. To accomplish that, we first need to establish reproducible and precise quality control of CHIP calls. Accurate classification of CHIP will enhance the approximations of their associations with (atherosclerotic) cardiovascular disease and allow clinicians and scientists to generate evidence-based algorithms for reporting thresholds.

Furthermore, the challenge lies in determining the appropriate cardiovascular surveillance and cardiovascular risk reduction (lifestyle, medication) strategies for this diverse group of individuals. Achieving this aim will require long-term studies of healthy individuals, incorporating extensive batteries of baseline and follow-up cardiovascular assessments, enriched by an array of circulating and imaging-based inflammatory and immune markers. Their use should help us understand which individuals with CHIP require follow-up, which should intensify lifestyle modification, and which might benefit from a pharmacologic risk-reducing intervention, such as statins, while providing insights into the role of predictive biomarkers and correlates of response. Validated risk scores of clinically relevant ASCVD in individuals newly found to have CHIP, similar to those used to predict myeloid neoplasms (57), will be a key next step. Yet to capture a sizable number of cardiovascular events and have sufficient power, trial participants will have to be monitored for many years, a technically and logistically challenging endeavor.

The detection of CHIP also introduces a potential for harm in the form of downstream overtesting and anxiety about next steps and one’s future health, which is particularly relevant in the absence of proven interventions that improve outcome. While lifestyle modification is a given that can be adopted in most cases, initiation of medications such as aspirin entails some risk. Although ideally, convincing data would inform implementation, in the short term we may need to act, weighing the risks and potential benefits in an informed and shared decision-making mode with individuals with CHIP. Trials for novel therapies, however, are difficult to conduct: each CHIP mutation is distinct and has a defined variant frequency with likely varying kinetics, some individuals have more than one mutation, and there might be interactions between nongenetic cardiovascular risk factors and some CHIP mutations. Clinical investigations should therefore start with higher-risk subjects with CHIP, such as those harboring *TET2* or *JAK2* mutations at high allele frequency (>10%), those with several mutations at high allele burden, and those with preexisting very high cardiovascular risk.

In addition, while at present, the detection of CHIP occurs in the course of evaluation of possible hematologic malignancy, during molecular analysis of a solid neoplasm, or after hematopoietic stem cell transplantation, trials will have to expand eligibility algorithms since the overarching goal is to learn about the benefits of atherogenesis prevention or modification in cohorts with normal projected life expectancy, rather than solely in patients with cancers who may not survive long enough to manifest clinical ASCVD. Finally, enrollment in trials should strive to achieve diversity in sex, ethnicity, and socioeconomic backgrounds to promote equity as we move to further untangle the complex web of CHIP and ASCVD.

## Figures and Tables

**Table 1:**
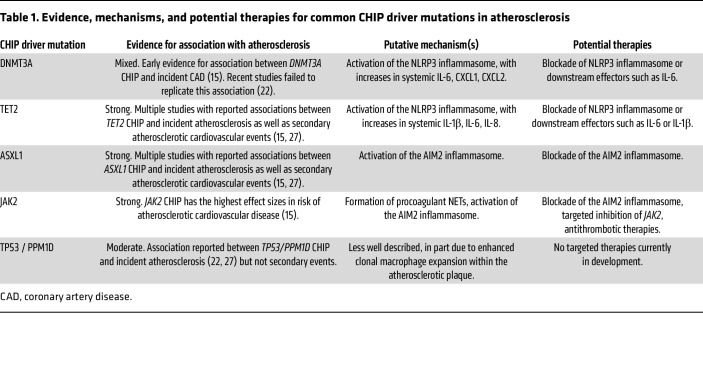
Evidence, mechanisms, and potential therapies for common CHIP driver mutations in atherosclerosis

## Version 1 10/01/2024

Electronic publication
